# Post-fire invasion risk of Chinese tallow (*Triadica sebifera*) in a slash pine flatwood ecosystem in the Gulf of Mexico Coastal Plain, United States: mechanisms and contributing factors at the community level

**DOI:** 10.48130/FR-2021-0002

**Published:** 2021-01-22

**Authors:** Nannan Cheng, Zhaofei Fan, Nancy Loewenstein, Robert Gitzen, Shaoyang Yang

**Affiliations:** School of Forestry and Wildlife Sciences, Auburn University, Auburn, AL 36849, USA

**Keywords:** Chinese tallow, slash pine flatwood, fire, dispersal factors, community factors

## Abstract

The invasion of Chinese tallow (*Triadica sebifera* (L.) Small) is a serious threat to the endangered slash pine (*Pinus elliottii*) flatwood ecosystem in the Gulf of Mexico Coastal Plain, United States. Prescribed fire in combination with vegetation management has been suggested as a preferred approach for mitigating Chinese tallow invasion and restoring this endangered ecosystem. A large plot of 0.86-ha with 281 nested contiguous 30-m^2^ quadrats was established in a tallow-invaded slash pine flatwood and all tallow trees, saplings, seedlings and associated factors in each quadrat were measured to study the community-level tallow invasion processes before and after a prescribed fire and by dispersal and community factors. Classification and regression tree models show that the dispersal factors (distances to the road and to the trail) and microtopography (elevation) determine the invasion probability of tallow, but the degree of invasion (abundance) of tallow depends on the interactions of both dispersal factors and community factors such as canopy closure and grass/herbaceous coverage. Areas nearer to roads and trails, dominated by native grass/herbaceous species, and with a low elevation and canopy closure are highly susceptible to tallow invasion and establishment. The effect of fire on tallow invasion varies with overstory and understory conditions. Density of tallow seedlings and saplings increased greatly after fire in the areas dominated by slash pines in the overstory and native grass/herbaceous species in the understory. To control tallow invasion and establishment, tallow seed trees/sources should be removed from the area and vicinity to be burned.

## INTRODUCTION

Chinese tallow (*Tricdica sebifera*, (L.) Small, hereafter, tallow), which is native to Japan and central China, was introduced into the southern region of the United States in the late 1700s as an ornamental and potential oil species^[1,2]^. In the 1900s, the U.S. Department of Agriculture promoted the planting of tallow for the production of industrial oils in the Gulf of Mexico coastal region^[3,4]^. Since then, tallow populations have rapidly spread across diverse ecosystems and landscapes in this region. By the early 2000s, tallow had occupied 185,000 ha of forestlands in east Texas, Louisiana, and Mississippi^[5,6]^. Based on the U.S. Department of Agriculture Forest Service’s Forest Inventory and Analysis (FIA) data, Wang et al.^[7]^ estimated that tallow could occupy more than 1.5 million ha of forests by 2023. An analysis of the most recent FIA data from across the 67 coastal counties of northern Florida, Alabama, Mississippi, Louisiana and eastern Texas showed that tallow has outcompeted many native species, ranking 17^th^ out of the 135 encountered species in aboveground dry weight^[8]^. To date, tallow has become one of the most prevalent invasive species in many native ecosystems, threatening not only native species composition and community structure, but also transforming soil properties, ecological processes, and ecosystem functionalities and services^[9-11]^.

Adaptability of an invasive species like tallow to new environments is an important factor for successful invasion^[12,13]^. Similar climate and forest conditions to its native range in the Gulf of Mexico coastal region are favorable for tallow invasion^[7,14]^. The advantageous physiological characteristics and life history traits of tallow such as seedling shade and flood tolerance, long seed dormant season, large leaf area, quick nutrient uptake, strong sprout capacity, rapid growth rate, and herbivore resistance make it able to dominate recipient ecosystems rapidly^[14-18]^. Furthermore, recurrent, large-scale disturbances such as hurricanes, tropical storms, fires, and resultant changes in environmental conditions significantly facilitate its colonization, establishment, and spread^[19-21]^.

The slash pine (*Pinus elliottii*) flatwood is an endangered ecosystem highly susceptible to tallow invasion due to its open overstory structures and hydrologic condition which includes a frequent flooding regime^[22]^. Historically, it occupied broad areas of the Gulf of Mexico coastal plain and provided critical ecological functions and services, but is now reduced dramatically in many areas due to large-scale exploitable land use change and fire suppression^[23]^. Prescribed fire combined with vegetation management has been suggested as a potential management tool to restore this endangered ecosystem and control tallow invasion, but has not been tested experimentally^[23-25]^. Frequent growing-season burning can kill tallow seedlings, damage saplings and curb tallow establishment, yet fire disturbances may consume understory vegetation and litter cover, promote seed germination and increase tallow invasion risk due to increased resource availability^[10,23,24]^. Understanding of fire effects and trade-offs currently is incomplete due to the differences in fire regimes (e.g., seasonality, intensity, and frequency) and/or varying ecosystem conditions tested^[10,11]^.

With increasing threats from tallow invasions, prescribing effective control and mitigation treatments requires an understanding of the mechanisms of how biotic and abiotic factors across varying spatial and temporal scales promote or impede tallow invasion in order to prescribe effective control and mitigation treatments^[22,23]^. While recent studies have examined spatial variations and contributing factors of tallow invasions on a meso- or large-scale, from a landscape to a geographic region^[20,21,26]^ , information about invasion processes and associated factors at finer local- or micro-scales is lacking. The poor understanding of finer scale invasion processes has become a barrier to predicting ecosystem responses and prescribing treatments for the restoration of endangered native ecosystems and control of biological invasions^[27]^.

In order to capture tallow’s spatiotemporally hetero-geneous invasion processes^[21-23]^ at microscales (e.g., within a plot or stand), we selected a large (~0.9 ha) plot in an invaded slash pine flatwood. This stand varied spatially in both overstory and understory conditions and was partially burned by a wildfire in 2016 and a prescribed fire in 2019, providing an ideal condition to evaluate the effects of microscale stand and site factors on tallow invasion following fire disturbances. We noted that few studies considered the interactions of contributing factors across space and over time explicitly during sampling and field data collection^[28-31]^, which may result in the findings and results derived being less useful in prescribing control and mitigation treatments. Therefore, we differentiated tallow populations into three size (age) classes (trees, saplings and seedlings) and mapped them in a grid of contiguous quadrats to maintain the integrity of data for studying the spatiotemporal invasion processes. In this way, the interactions of potential contributing factors and tallow invasion processes could be examined explicitly in space and over time. Specifically, this study addressed the following two questions/objectives: 1) How do associated factors affect tallow’s invasion probability and degree at the community level? 2) How does tallow of varying sizes (developmental stages) respond to recurrent, low-intensity ground fires? This information is critical to prescribe fire and silvicultural treatment for the restoration of the endangered slash pine flatwood and mitigation of tallow invasions.

## MATERIALS AND METHODS

### The conceptual model of tallow invasion following fire at the community level

**Figure 1:**
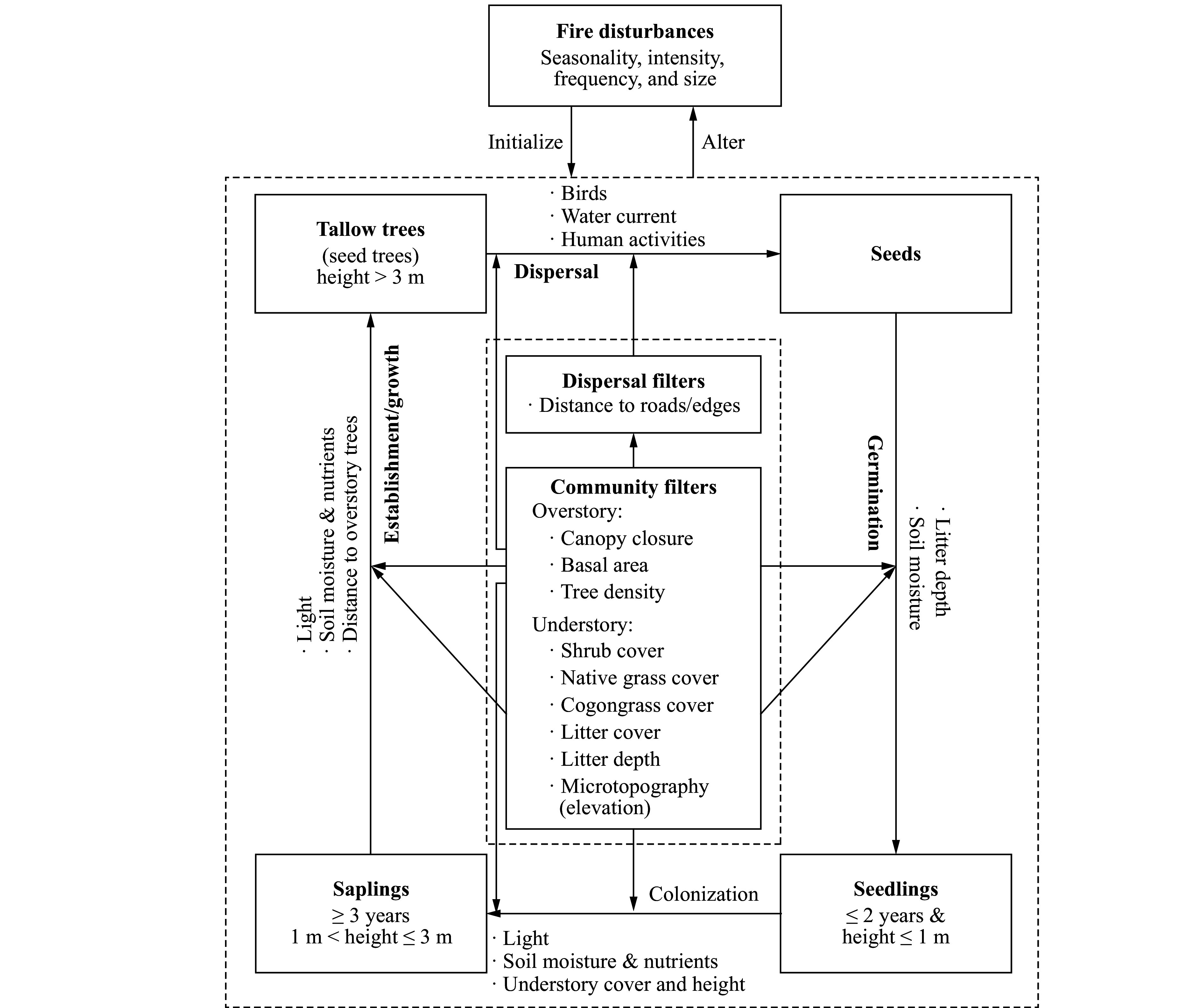
The conceptual diagram of tallow invasion at the community level showing the filters that affect tallow invasion processes. Tallow invasion includes four stages (seed dispersal, seed germination, seedling colonization, and sapling establishment/growth into tallow trees) with major corresponding limiting factors (bulleted) (adapted from Fan et al. 2021^[37]^).

Fire (wild or prescribed) is a driving factor of tallow invasion and may initiate and alter the invasion processes by changing resource allocation^[23,27]^. Fire can clear understory vegetation and promote seed germination and seedling recruitment, but recurrent fires, especially those frequent, low-intensity fires may kill or top-kill small tallow trees before they grow into larger sizes that are more resistant to fire (Fig. 1). The effect of fire on tallow invasion depends not only on the invasion stage (propagule pressure level, tree age and size) and fire regime (seasonality, intensity, and frequency) but also on community conditions^[10,23,25,36]^. The invasion probability and abundance of tallow in a fire-managed community may vary significantly with site/community characteristics and propagule pressure levels and need to be evaluated for varying community conditions to develop effective prescribed fire treatments to control and mitigate tallow invasion.

Specifically, tallow invasion processes and dynamics at the community level are influenced by a set of filters (factors) including both the extrinsic dispersal filters and the intrinsic community filters that may facilitate or impede the invasion process^[32]^. With the disturbance-dependent tallow, the extrinsic seed/propagule dispersal processes are mainly controlled by the spatial distribution of seed trees (sources), landscape structures (dispersal pathways such as edges and corridors), and disturbances (driving water current, birds, and energy and matter flow), which determine the likelihood for a community to be invaded. On the other hand, community filters measure intrinsic invasion processes (seed germination, seedling colonization, sapling establishment and growth into the overstory) and determine community invasibility (susceptibility) and the realized invasion outcomes such as spatiotemporal patterns and degree of invasion^[28,31-33]^. Potential community filters include overstory condition (e.g., canopy closure (%), tree density, and species composition) and understory condition (e.g., microtopography (elevation), litter coverage and depth, and species composition such as the coverage (%) of different life forms such as shrubs, grasses/herbaceous species, and vines). Overstory condition may directly affect light condition and litter depth, which can directly affect seed germination and seedling growth, and indirectly affect seed distribution by regulating bird activities^[34,35]^. Understory conditions such as microtopography (elevation) and vegetation can affect soil moisture, seed germination, and the competition between tallow germinants and native species. Although numerous factors, boitic and abiotic, contribute to tallow invasion, this study will focus on evaluating how commonly managable, biotic community factors affect tallow invasion (objective 1) and ecosystem invasibility (objective 2) following fire (Fig. 1).

### Study sites

**Figure 2:**
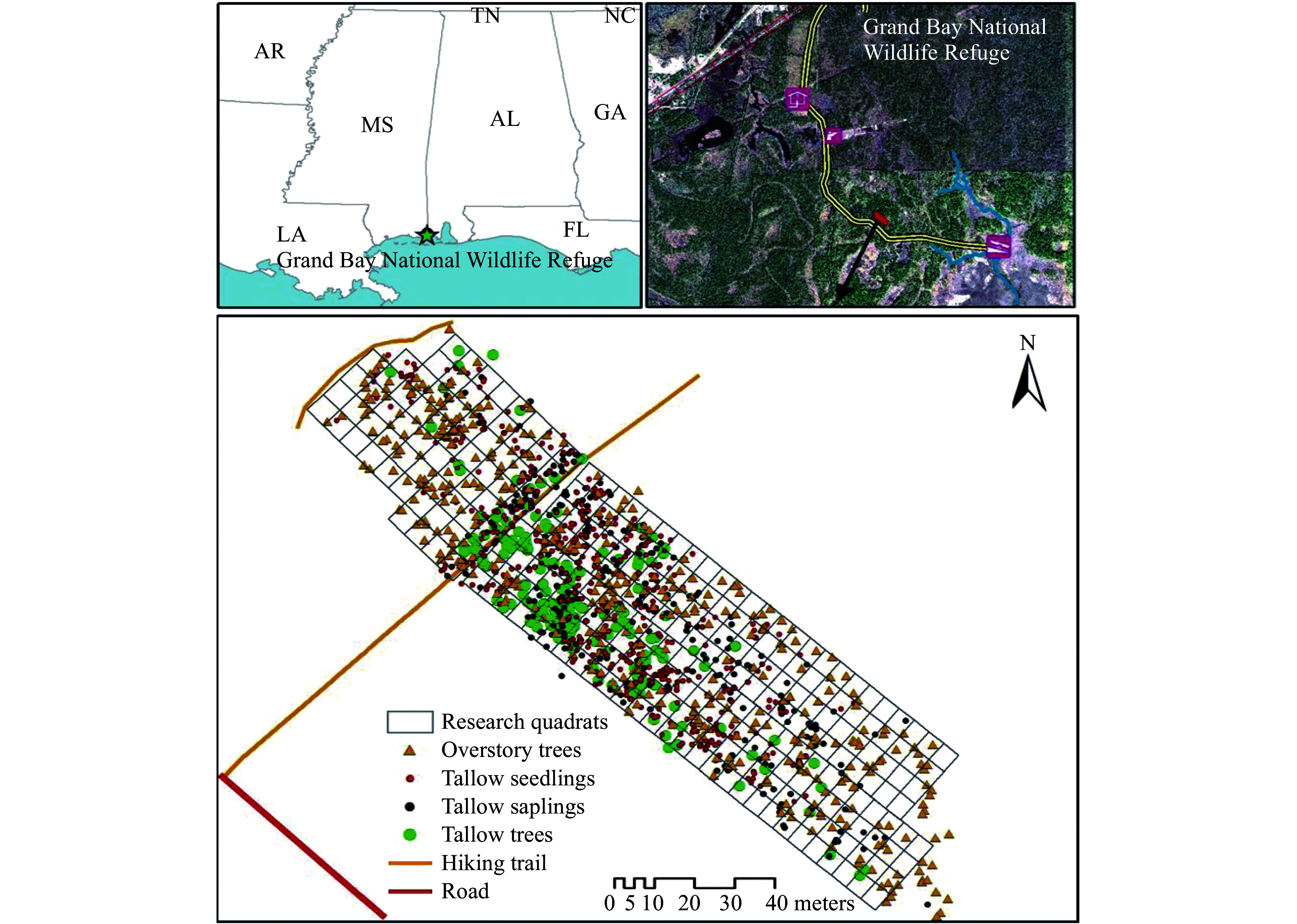
The invasion pattern of tallow populations in the 0.86-ha plot (281 contiguous 30-m^2^ quadrats) in the slash pine flatwood in the Grand Bay National Wildlife Refuge.

The Grand Bay National Wildlife Refuge (GBNWR) was established in 1992 with a primary objective of restoring wet slash pine flatwoods. Located on the Mississippi/Alabama state line in Jackson County, MS (30°25'12" N, 88°25'12" W) (Fig. 2)^[22]^, GBNWR is situated within the deltas of the Escatawpa and Pascagoula rivers, and is in the lower Gulf Coastal Plain. The total area of the refuge is 7,285 ha, and 81% of the area is public lands and waters. The major cover types in this area are wet pine flatwoods and savannas, along with salt marshes, maritime forest, wetlands, and bays. The climate of GBNWR is a subtropical climate with hot and humid summers. The average annual maximum and minimum temperatures are 24.7 °C (76 °F) and 14.7 °C (58 °F), respectively. The annual rainfall is around 1.6 m, and extreme rainfall events can lead to serious riparian flooding^[38]^. The invasion of tallow to GBNWR is thought to have originated from plants located on private inholdings and around roads and boundaries since the 1990s^[39]^. In March 2016, a wildfire swept over a large part of the slash pine flatwood in the refuge, including the area where this study was conducted. In May 2019, a low-intensity, prescribed fire was burned in the study site and vicinity. Recent tallow invasion episodes were observed following fire disturbances.

### Data collection

In March 2018, a study plot of 0.86-ha in a wet pine flatwood was established. A hiking trail, with clumps of tallow distributed along either side, passes through the plot (Fig. 2). All overstory trees (native and tallow trees) were mapped using a high-resolution GPS (Global Positioning System) unit with tree species and diameter at breast height (dbh) being recorded and measured^[22]^. The plot was then divided into 281 contiguous 30-m^2^ quadrats to measure tallow invasion as well as overstory and understory conditions.

First, all measured tallow trees were divided into three size classes: tallow trees (> 3 m in height), saplings (1 m < height ≤ 3 m) and seedlings (height ≤ 1 m), and the number of tallow by size class and number of total overstory trees (including pine and hardwoods) were counted for each quadrat. Within each quadrat, thirty evenly-placed 1 m^2^ sub-quadrats were further set up to record the vegetation types: cogongrass (*Imperata cylindrica* (L.) Raeusch), native grass, shrub, litter, pine sapling. Based on the records of the 30 sub-quadrats of each quadrat, we calculated the coverage (%) of cogongrass, shrub, and native grass. For the litter depth, we set up 5 points in the 30-m^2^ quadrat to measure the depth and calculated the mean value. For the height of the pine saplings, we measured the height of all saplings and calculated the mean value. We used a crown densiometer to measure the canopy closure (%) of each quadrat. The distance from each quadrat to a tallow-invaded road and hiking trail (seed sources) was also measured using ArcGIS. The digital elevation model (DEM, 3m resolution) was obtained from the United States Geological Survey (USGS) to show the topographic conditions of the study plot. The mean elevation (m) of each quadrat was extracted from the DEM to show the change of microtopography.x

In August 2019, the plot was remeasured after a prescribed burn in May 2019. In each 30-m^2^ quadrat, the number of tallow seedlings and saplings (including those re-sprouted and that grew from seedlings) were measured and recorded. We also remeasured the coverage and height of shrubs and grasses/herbaceous species as well as the coverage and depth of litter in each quadrat. Based on the dominant vegetation type or life form which was measured in coverage (%), each quadrat was classified into five substrate conditions: native grass (NG), pine seedlings and saplings (PS), shrubs (SB), cogongrass (CG), and litter cover (LC).

### Data analysis and modeling

To quantify tallow’s invasion probability and degree (abundance) in the slash pine flatwood and evaluate the effects of associated factors (objective 1), we first plotted the smoothed probability density function of tallow by size class and the bivariate scatterplots between size classes (tallow trees, saplings, and seedlings) based on the data collected from quadrats (n = 281). With the extremely right (positive)-skewed and zero-inflated (excessive zeros) count data of tallow, we applied the nonparametric classification and regression tree model (CART) to analyze quadrat data to quantify the effects of potential factors and their interactions on the invasion probability and degree (count) of tallow^[40]^. A quadrat was classified as tallow absence (0; non-invaded) or presence (1; invaded) categories based on whether or not tallow was found. Using the binary presence-absence variable as the response variable and the dispersal and community factors measured as the predictor variables, a CART model of invasion probability was constructed. To prevent overfitting, the CART model was pruned based on the complexity measure to achieve the minimum relative error. The evaluation of selected factors and invasion patterns were based on the pruned CART model (the best model). Likewise, using the count of tallow trees, saplings and seedlings in each quadrat as the response variable and the same set of factors as the predictors, three CART models were constructed and pruned, respectively, to evaluate the patterns of the degree of invasion by significant factors and interactions. R packages *rpart* and *rpart.plot* were used for developing and plotting the CART models^[40,41]^.

To address how tallow invasions respond to fire (objective 2), analysis of covariance and post-hoc Tukey multiple comparisons were conducted to evaluate post-fire change in the number of tallow seedlings and saplings (recent invasions) by understory conditions (a factor of five levels: NG, PS, SB, CG, and LC) and covariates including the coverage of shrubs, grass/herbaceous species and litter. All analyses were done using the R language^[41]^ and a priori significance level of α = 0.05 was set for statistical tests.

## RESULTS

### Effects of risk factors on tallow invasion at the community level

**Figure 3:**
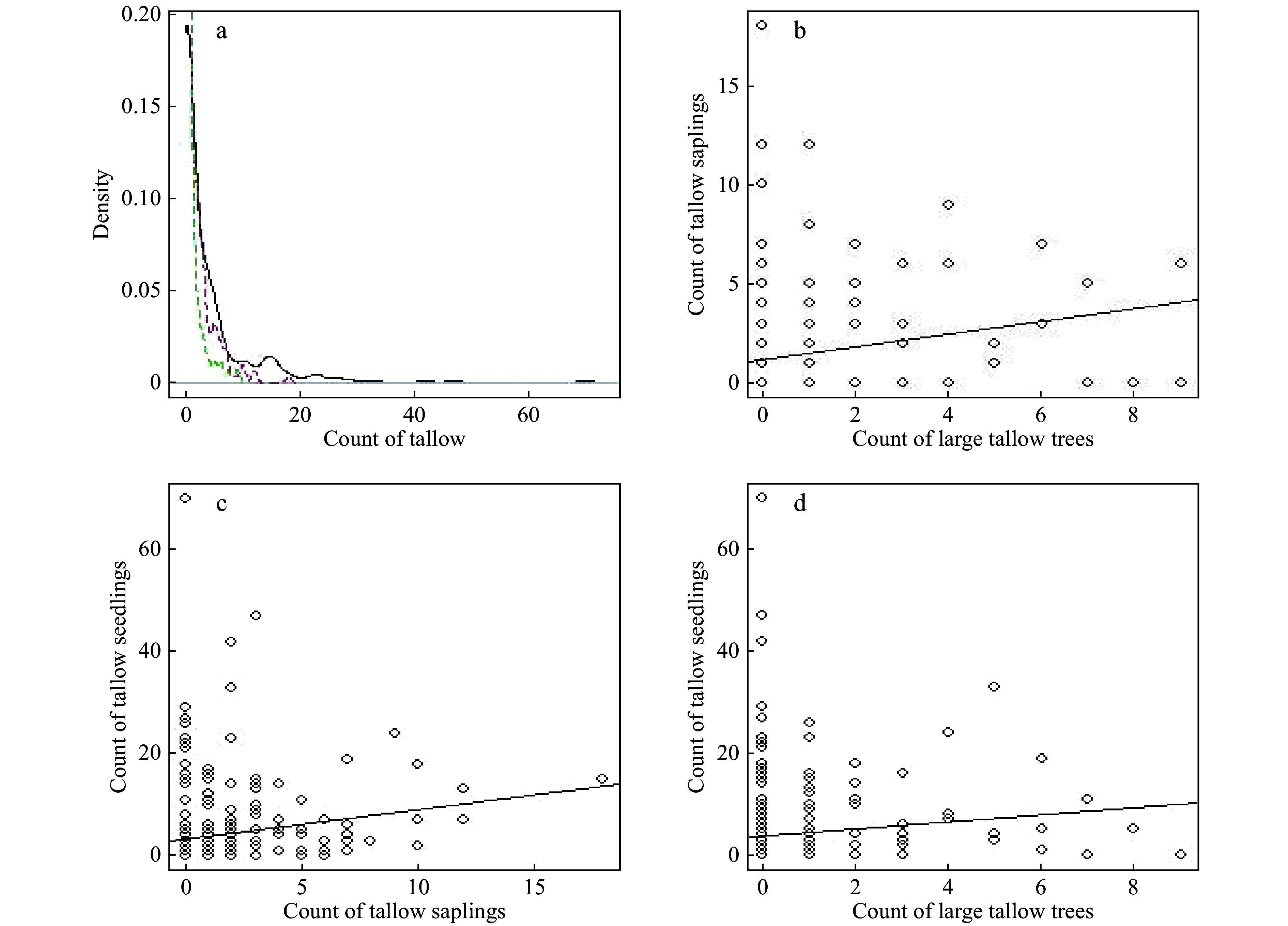
The smoothed probability density function (a, green dashed line: tallow tree, red dashed line: saplings, black line: seedlings) and the scatterplots (b-d) of tallow count in each quadrat by size class. The straight line represents the linear trend line.

Within the study plot, 66 (23.5%) out of 281 quadrats were invaded by tallow. The number of invaded tallow (large trees, saplings and seedlings) in the 30-m^2^ quadrats followed a skewed-to-right distribution with a mean of 0.6 large tallows, 1.4 saplings and 4.1 seedlings per quadrat, respectively (Fig. 3a). Although there was a positive relationship (trend) between tallow size classes (large tree vs saplings, large tree vs seedlings, saplings vs seedlings), great variations among quadrats existed. This suggested tallow invasion was controlled by multiple factors and changed across both space and measurement time (Fig. 3b, c and d).

**Figure 4:**
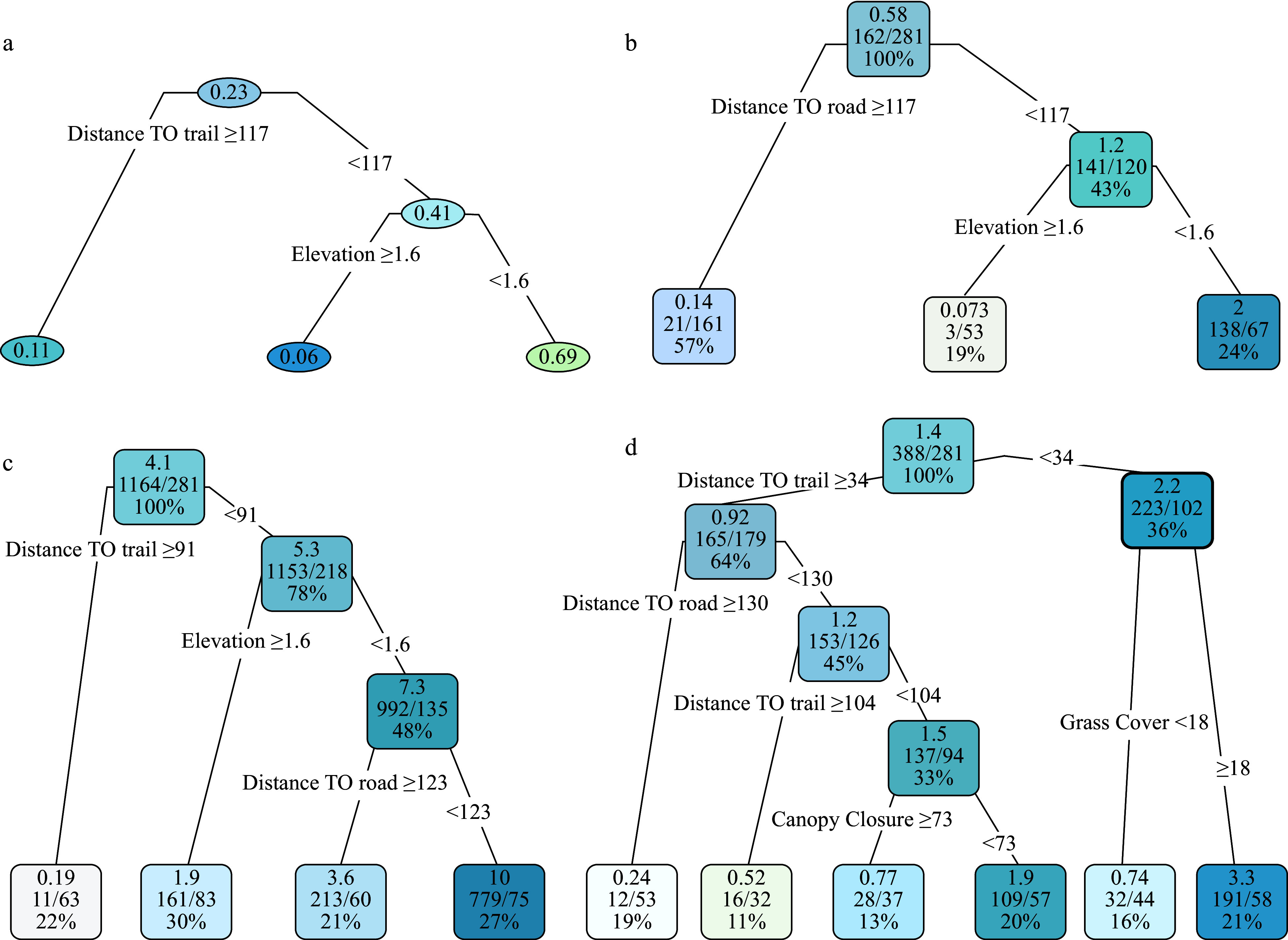
The presence probability (a) and invasion degree CART models (b-d) for tallow by size class (b: large tallow; c: seedling; d: sapling). The number in each node in Fig. a represents invasion probability among the quadrats that meet the splitting criteria. The top, middle and bottom numbers in each node in Fig. b-d represent the average number of tallow trees in a quadrat, total number of tallow trees/total number of quadrats, and the proportion of quadrats falling within a node.

The probability for a site (a quadrat) to be invaded by tallow was primarily influenced by two factors: distance to the road (a proxy of propagule pressure) and elevation (Fig. 4a). Quadrats < 117 m from the road had an invasion probability of 0.41, which was 3.7 times higher than quadrats ≥ 117 m (invasion probability = 0.11). Within the areas nearer (< 117 m) to the road, the average invasion probability reached up to 0.69 for quadrats < 1.6 m in elevation, which was 11.5 times higher than quadrats ≥ 1.6 m in elevation. The CART model for the abundance (degree of invasion) of large tallow trees was equivalent to the invasion probability model in significant variables and their cutoff values (Fig. 4b). The total number of large tallow trees was 162 among the 281 quadrats, and approximately 87% (141/162) of large tallow trees were found within the 120 quadrats < 117 m from the road, with the remaining 13% (21/162) occurring within the 161 quadrats ≥ 117 m from the road. Among those quadrats < 117 m from the road, nearly all large tallow trees (97.9%, 138/141) were found in quadrats with low (< 1.6 m) elevation (Fig. 4b).

Recent tallow invasions (seedlings and saplings) continue to be confined to previous invasion areas (areas with large tallow trees) and vicinities. The colonization and establishment of tallow saplings and seedlings were mainly controlled by the distance to the hiking trail (Fig. 4c and d). Other variables including distance to the road, elevation, canopy closure and grass coverage appeared to be important as well. Quadrats with a shorter distance to the road, lower elevation, smaller canopy closure, and larger grass cover (lower shrub cover) had more tallow saplings and seedlings (Fig. 4c and d). Generally, more tallow saplings and seedlings were found in quadrats with shorter distances to the road and the trail, lower elevation and canopy closure, but higher grass coverage.

### Post-fire changes of tallow saplings and seedlings by overstory and understory vegetation conditions

**Figure 5:**
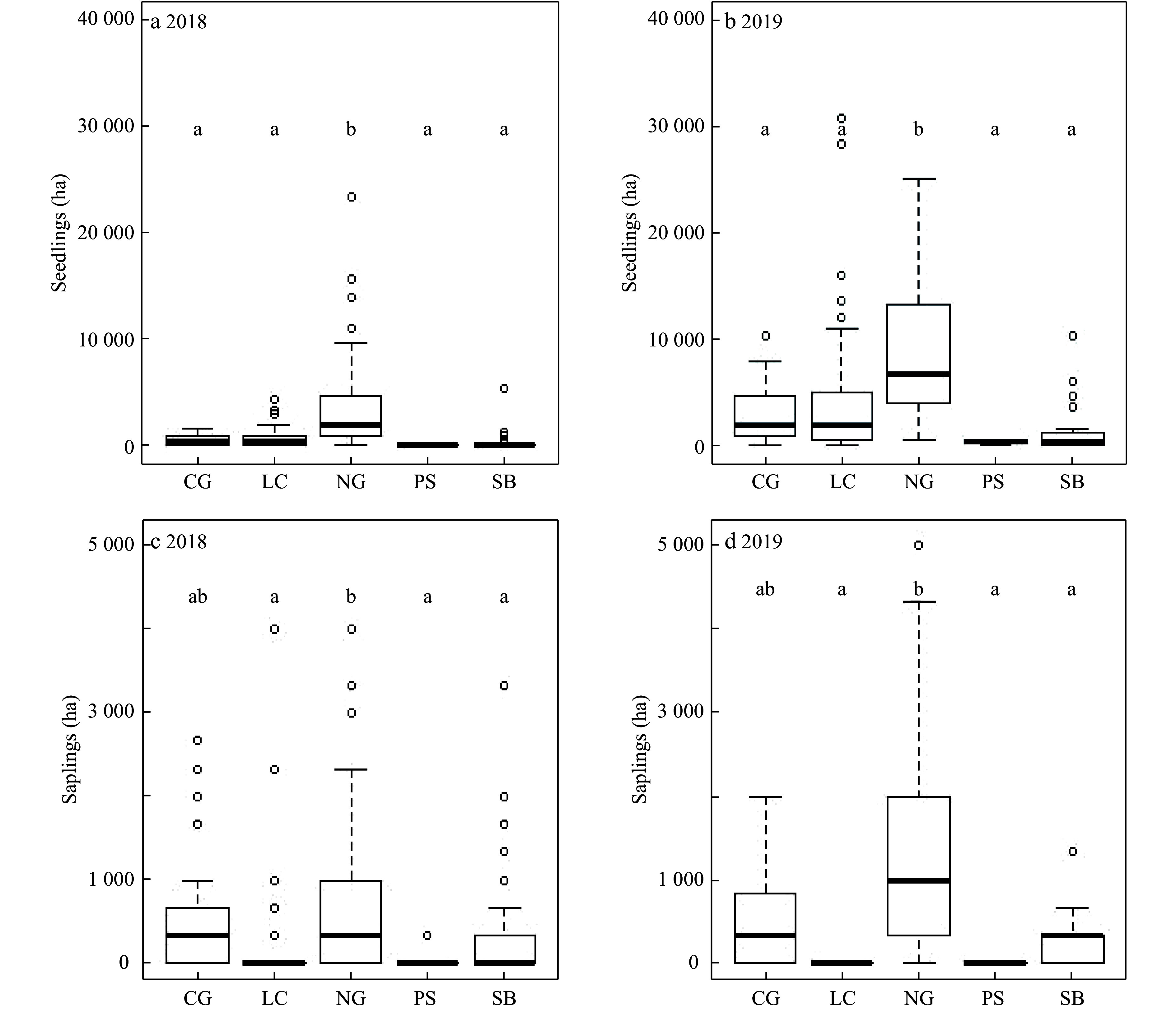
Changes of tallow seedlings and saplings by understory vegetation cover type (CG=cogongrass, LC=litter covered, NG=native grass, PS=pine sapling, and SB=native shrub) before (2018) and after (2019) the prescribed burn in the slash pine flatwood in the Grand Bay National Wildlife Refuge.

The area dominated by native grasses had the most tallow seedlings before (2018) and after the prescribed burn (2019). With the exception of the areas dominated by pine seedlings and saplings, the number of tallow seedlings increased significantly in all vegetation types after the burn (p < 0.05) (Fig. 5a and b). The number of tallow saplings in native grass-covered areas was also greater than that of other vegetation-covered quadrats except the cogongrass-covered areas in 2018 (Fig. 5c) and 2019 (Fig. 5d).

**Figure 6:**
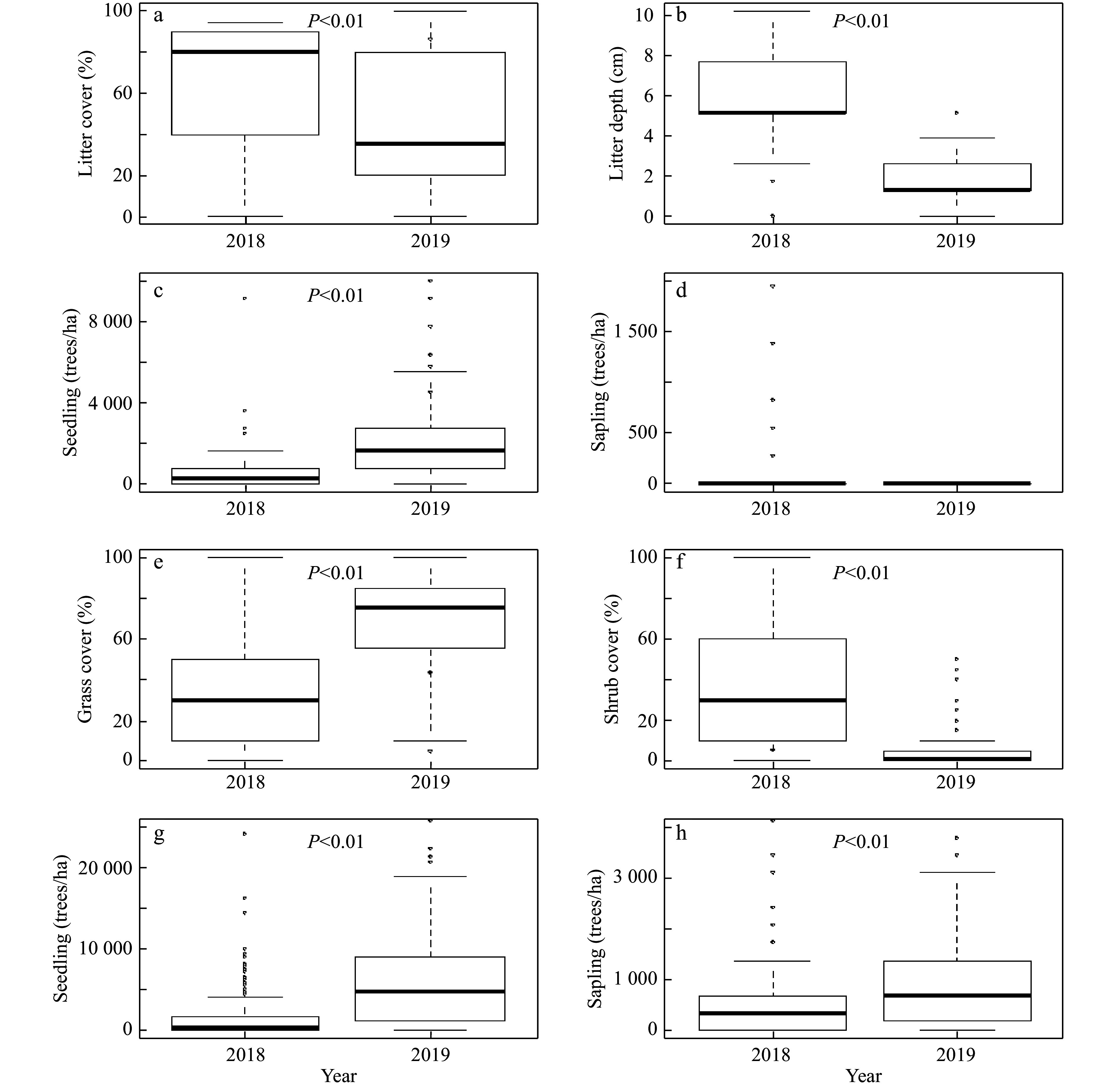
Changes of understory vegetation conditions and tallow seedlings and saplings where hardwoods dominated the overstory (a-d) and where pines dominated the overstory (e-h) before (2018) and after (2019) the prescribed burn in the slash pine flatwood in the Grand Bay National Wildlife Refuge.

Further analyses of understory conditions and tallow invasion data in light of overstory conditions showed that the increase in tallow seedlings in the hardwood-dominated areas following the prescribed burn was significantly correlated to the decrease in litter cover and depth. The increase in the number of tallow seedlings following the prescribed burn was statistically significant, but the number of tallow saplings was nearly the same before and after the burn (Fig. 6a-d). In the pine-dominated areas, both tallow seedlings and saplings increased significantly following the burn. Meanwhile, a significant increase in the coverage of grass/herbaceous species, but decrease in the coverage of shrubs was observed following the burn (Fig. 6e-h).

## DISCUSSION

The extremely right-skewed probability density functions and positive trends between the number of tallow trees, saplings and seedlings among the 281 contiguous quadrats provided strong evidence of clustered invasion patterns or spatial coexistence of invaded tallow of different size classes by potential limiting factors to the invasion processes^[22,27,37]^. In accordance with the mechanistic model, the probability and abundance/degree of invasion of tallow are related to both dispersal filters and community filters (Fig. 1). The CART models (Fig. 4) explicitly show the critical role of dispersal filters (distance to roads and trails) and their interactions with community filters (elevation, canopy closure, and grass coverage) in tallow invasion processes^[22,23,26,37]^. The invasion probability of tallow as a whole (Fig. 4a) and the distribution of tallow trees and seedlings (Fig. 4b and c) are predominantly determined by factors directly related to seed dispersal by birds and water current. Areas close to forest edges (e.g., road, hiking trail, ditch, fire line) and/or with low elevation are more likely to be invaded by tallow because of high propagule pressure − a large soil seed bank stored up by birds and/or water current^[8,34,35]^. However, the establishment of tallow saplings (Fig. 4d) may depend on the interactions between dispersal filters and community filters.

As a pioneer or disturbance-dependent species, tallow establishment and growth depend on sufficient light, although tallow seedlings can tolerate a wide range of light conditions^[42-44]^. This explains why large numbers of tallow saplings can only be found under low or moderate canopy closure (< 0.7), whereas numerous seedlings can colonize under high canopy closure (≥ 0.7)^[27]^. The role of understory vegetation cover in tallow establishment (from seedlings to saplings) is due to the negative competition from native species for light, nutrient, water, and growing space, which results in a spatial pattern that large numbers of tallow saplings mostly occur in areas with high grass/herbaceous species coverage and low shrub coverage^[8,22]^. Therefore, efforts to create a sparse canopy structure and to restore grass-dominated understory vegetation will inevitably increase the risk for tallow invasion and establishment in areas with high propagule pressure. To mitigate or control the risk, proactive treatments to reduce the propagule pressure by removing seed trees is critical prior to overstory and understory treatments such as thinning and prescribed burning.

Prescribed fire plays an important role in nutrient recycling, regulating plant succession and maintaining wildlife habitat, and it is an essential tool to protect and restore native ecosystems in the southern US^[24,36,45]^. Prescribed fire is also applied as a tool to control invasive species^[23,46]^. However, the effectiveness of fire in terms of maximizing the diversity of native species and minimizing the invasion risk of nonnative invasive species may change with understory conditions or ecosystems. In this study, the spatial heterogeneity of understory vegetation was observed in species or life-form (e.g., shrub, grass, herbaceous species) compositions with the changes in the overstory and the microtopography. In litter-covered and grass/herbaceous-species-dominated areas, prescribed fire can greatly facilitate tallow invasion. Prescribed fire consumes large amounts of the litter and understory vegetation that can impede invasion by the tallow tree. As a result, frequent prescribed fires may not only promote the dominance of grass/herbaceous species (suppressing shrubs) but also increase the risk of tallow invasion^[23,24]^. In areas dominated by pine seedlings and saplings, shrubs, and invasive cogongrass, however, the effect of fire is limited, perhaps due to intense competition from other native or invasive species.

Before the 2019 prescribed burn, larger numbers of tallow saplings accumulated in quadrats within areas with pine-dominated overstories compared to areas with hardwoods-dominated overstories. These saplings origniated either from the 2016 wildfire or other disturbance events (e.g., flooding, animal activities) and most of them survived the low-intensity prescribed burn in 2019, remaining intact or resprouting after the top-kill. This explains why the number of tallow seedlings increased dramatically after the 2019 prescribed burn in both hardwood-dominated and pine-dominated areas, but the number of tallow saplings increased significantly only in pine-dominated areas (Fig. 6). Overall, the number of tallow saplings and seedlings in pine-dominated areas was higher than that in hardwood-dominated areas, which suggests that pine-dominated areas have lower resistance and are more susceptible to tallow invasion following the prescribed fire^[23,27]^. In the pine-dominated areas, less litter accumulated in the understory compared with the deeper litter layers observed in hardwoods-dominated areas, but fire can consume litter and create suitable conditions for tallow seed germination and seedling colonization.

The invasion risk (probability) and degree of tallow germinants in fire-managed ecosystems are closely related to fire regimes such as the mean fire return interval and ecosystem structures^[10,23,26]^. Fan et al. 2020^[37]^ differentiated theoretical (intrinsic) ecosystem invasibility from realized ecosystem invasibility or outcomes for invasive species management and decision making. The former is determined only by community filters that control the interactions between invaders and native species. On the other hand, the latter depends on both dispersal filters and community filters. Invasive species management should focus on managing dispersal factors and processes to reduce invasion risks prior to any management activities (e.g., prescribed burning, thinning of overstory and understory). The spatially clustered patterns of tallow invasion suggest that manipulating dispersal filters such as the eradication of tallow seed trees along roadways or in invaded stands to reduce the propagule pressure level is especially important to curb tallow invasion as the activities to restore the resilience and health of native ecosystems proceed.

## CONCLUSION

Tallow invasion is a serious threat to the endangered slash pine flatwood ecosystem in the Gulf of Mexico Coastal Plain. Prescribed fire is an important tool for restoring native ecosystems, but its effectiveness varies with the size of the invader and ecosystem conditions. The successful establishment of tallow, from seedlings to saplings and large trees, needs to overcome the barriers from both the overstory and understory such as low light intensity and intense competition from other species. Efforts to restore the sparse overstory structures and the understory vegetation dominated by diverse native grasses and herbaceous species of the slash pine flatwood ecosystem tends to increase the risk of tallow invasions. However, whether tallow invasions happen or not on a site is greatly driven by its current propagule pressure levels and the conditions of dispersal factors, that is, the distance to roadways and forest edges. The risk factors and their cutoff values estimated by the CART models can be used as a decision tool for evaluating potential tallow invasion risks.

Prescribed fire combined with the removal of overstory and midstory trees can be used as a management tool to restore the desired conditions of the slash pine flatwood ecosystem. The removal of tallow seed trees on the site and from the vicinity prior to prescribed burning and vegetation management is critical to prevent the post-disturbance invasion and spread. Frequent fires, if carefully prescribed, can kill tallow seedlings and deplete soil seed bank. In order to design effective fire treatments for ecosystem restoration and control of invasive species such as tallow, multilevel (community, landscape, and geographic region) fire experiments need to be conducted in the future to test ecosystem responses and interactions between tallow and native species under different fire regimes.
